# Therapeutic Targeting of Lewis^y^ and Lewis^b^ with a Novel Monoclonal Antibody 692/29

**DOI:** 10.1371/journal.pone.0054892

**Published:** 2013-02-08

**Authors:** Philip Noble, Ian Spendlove, Stephen Harding, Tina Parsons, Lindy G. Durrant

**Affiliations:** Academic Department of Clinical Oncology, City Hospital Campus, University of Nottingham, Nottingham, United Kingdom; Technische Universitaet Muenchen, Germany

## Abstract

**Background:**

Several monoclonal antibodies (mAbs) recognising Lewis^y^, such as BR96, have reached the clinic but have failed to show good anti-tumour responses with an acceptable level of toxicity. No Lewis^b^ mAbs have been trialled in patients. In this study we compare the specificity of three mAbs; BR96 (Lewis^y^), 2-25 LE (Lewis^b^) and 692/29 that recognises a unique facet of both Lewis^y^ and Lewis^b^. We then assessed the *in vivo* therapeutic effect of 692/29 using xenograft models.

**Methodology/Principal Findings:**

Using a glycan array, each mAb was shown to display a different binding pattern with only 692/29 binding to both Lewis^y^ and Lewis^b^. 692/29 was able to kill tumour cells over-expressing Lewis^y/b^ directly, as well as by antibody and complement mediated cytotoxicity (ADCC/CDC), but failed to kill cells expressing low levels of these haptens. In contrast, BR96, directly killed cells expressing either high or low levels of Lewis^y^ perhaps explaining its toxicity in patients. 2-25 LE failed to cause any direct killing but did mediate ADCC/CDC. Both 692/29 and BR96 bound to >80% of a panel of over 400 colorectal tumours whereas 2-25 LE showed lower reactivity (52%). 692/29 demonstrated more restricted normal tissue reactivity than both BR96 and 2-25 LE. 692/29 anti-Lewis^y/b^ mAb also showed good *in vivo* killing in xenograft models.

**Conclusions/Significance:**

MAbs targeting both Lewis^y^ and Lewis^b^ may have a therapeutic advantage over mAbs targeting just one hapten. 692/29 has a more restricted normal tissue distribution and a higher antigen threshold for killing which should reduce its toxicity compared to a Lewis^y^ specific mAb. 692/29 has an ability to directly kill tumours whereas the anti-Lewis^b^ mAb does not. This suggests that Lewis^y^ but not Lewis^b^ are functional glycans. 692/29 showed good anti-tumour responses *in vivo* and is a strong therapeutic candidate.

## Introduction

The Lewis y and Lewis b antigens are tetrasaccharides and are extensions of the H blood group galactose-glucosamine. Although both are mostly expressed throughout foetal development and are gradually lost after birth, Lewis y and Lewis b can be expressed on normal cells in the gastrointestinal tract at low levels [1], [2]. Tumours have been shown to overexpress both Lewis y and Lewis b [3], [4], [5], making them good targets for mAb therapy. Lewis^y^ and Lewis^b^ glycans can be expressed on glycoproteins and glycosphingolipids (GSLs). Lewis^y^ has been shown to be associated with functional molecules that can effect tumour cell growth, adhesion, metastasis and proliferation [6].

The IgM mouse mAb, C14 mAb was raised against primary colorectal tumour cells using standard fusion protocols and binds to both Lewis^y^ and Lewis^b^ (extended and non-extended) antigens [7], [8], [9]. The Lewis^y^ hapten is a difucosylated tetrasaccharide (Fucα1-2Galβ1-4(Fucα1-3)GlcNAc) found on type 2 blood group oligosaccharides. This antigen is a positional isomer of the Lewis^b^ hapten (Fucα1-2Galβ1-3(Fucα1-4)GlNAc) and a fucosylated derivative of the Lewis^x^ hapten [4], [5]. The C14 mAb bound to 78% of colorectal cancers [10], but as a murine IgM it was unsuitable for *in vivo* studies. To produce an IgG variant of the antibody rats were immunised with C14 mAb and purified rat anti-C14 produced. Immunisation of mice with this antiserum and C14 affinity purified glycoprotein, followed by the fusion of their splenocytes with a mouse myeloma resulted in the production of five IgG mAbs; two IgG3s (692/23, 692/29 mAb) and three IgG1s (692/33, 692/42 and 692/43). All of the IgG variants recognised the Lewis^y^ and Lewis^b^ antigens and demonstrated the same specificity as C14 mAb [9]. Furthermore, these antibodies were shown by thin layer chromatography and ELISA to bind to extended and non-extended Lewis^y^ and Lewis^b^ haptens, but not to Lewis^x^ or H blood group haptens [9].

MAbs to Lewis^y^ and Lewis^b^ have been produced previously. Examples of Lewis^y^ mAbs that have been assessed for therapeutic value are BR96 and hu3S193 [11], [12], [13]. 692/29 mAb variants are novel as they recognise both Lewis^y^ and Lewis^b^ determinants. Only one rare lectin recognises a similar facet of these two molecules [14]. Crystallographic studies have shown that mAbs specific to Lewis^y^ can have very different binding sites which accommodate either the N-acetyl-glucosamine or the fucose residues within the hapten [15]. 692/29 is different again as its binding site accommodates an aspect common to both Lewis^y^ and Lewis^b^, which is very unusual as they are stereoisomers of each other. A recent study analysed a number of mAbs binding to the pancreatic cancer marker, CA19.9, and highlighted the variability between mAbs directed at similar glycan targets [16].

In this study we compare the therapeutic potential of Lewis^y^, Lewis^b^ and Lewis^y/b^ mAbs *in vitro* and test the *in vivo* efficacy of 692/29. The hypothesis is that a mAb recognising both haptens may have an improved therapeutic profile when compared to the more hapten specific mAbs.

## Results

### Defining the Epitopes Recognised by the Lewis^y/b^ Antibodies Using the Consortium for Functional Glycomics Glycan Array

**Figure 1:**
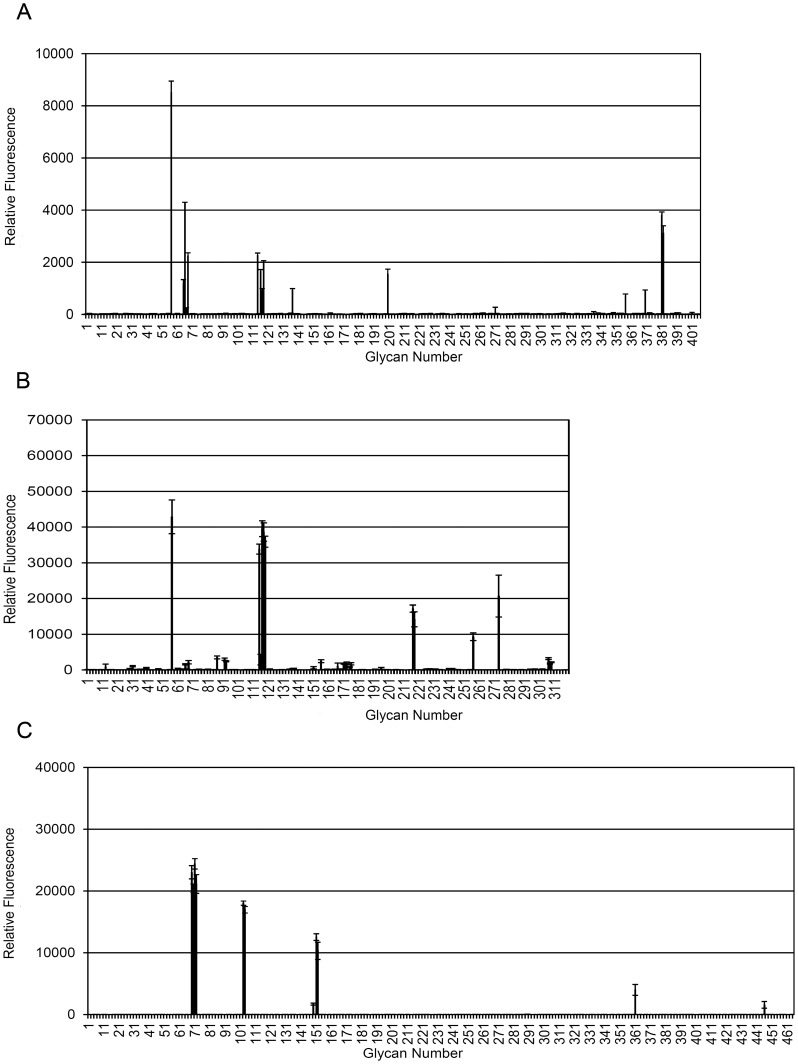
The fine specificity of binding of anti-Lewis^y^ and Lewis^b^ mAbs. The binding of 692/29 (A), 2-25 LE (B) and BR96 (C) to the Consortium for Functional Glycomics glycan array. Where a = Lewis^a^, b = Lewis^b^, y = Lewis^y^, x-Lewis^x^, D- = Di, T- = Tri, S- = sialyl, Ex- = extended, φ denotes a mannose containing glycan, and y×x = Lewis^y^-Lewis^x^.

Studies have shown that due to the subtleties observed in antibody binding to tumour cells and the high level of similarity between some carbohydrates, including blood group antigens, many antibodies have been found to cross-react with other related carbohydrates. The recognition of Lewis^y^ as a good therapeutic target has led to the production of a number of anti-Lewis^y^ mAbs. Despite being presented as Lewis^y^ specific mAbs, some have been shown to cross-react with other Lewis antigens. In order to clarify their fine specificity, the anti-Lewis^y/b^ mAb, 692/29 and the anti-Lewis^y^ mAb, BR96, were assessed and compared to the published specificity data for 2-25 LE (an anti-Lewis^b^ mAb) from the Consortium for Functional Glycomics. Each mab is screened independently and the binding patterns but not the absolute fluorescence can be compared. The relative binding of 692/29 to the array showed that the mAb bound most strongly to Lewis^b^, glycans containing Lewis^b^ and also to tri-Lewis^y^ and its variants (Fig. 1A). Analysis of the 2-25 LE binding data showed strong binding to Lewis^b^, with cross-reactivity to Lewis^a^ and Lewis^a–x^. Weak binding could also be seen against sialyl Lewis^a–x^, with no binding to Lewis^y^ (Fig. 1B). BR96 showed strong binding to Lewis^y^ as well as to a range of Lewis^y^ variants (Lewis^y–x^, Lewis^y-x-x^) and more weak binding to Lewis^x^. BR96 did not bind to Lewis^b^ (Fig. 1C). A representation of the glycans bound most strongly is displayed in Table 1 (a complete list of glycans bound by each mAb can be found in the supplementary data Table S1).

**Table 1:** Lewis^y/b^ mAbs have differing fine specificity. 692/29, 2-25 LE and BR96 were screened on the Consortium for Functional Glycomics glycan array.

Antibody	Glycan No.	Nomenclature	Name	PercentageFluorescence (%)
**692/29**	57	Fuca1-2Galb1-3(Fuca1-4)GlcNAcb-Sp8	Lewis^b^	**100**
**692/29**	66	Fuca1-2Galb1-4(Fuca1-3)GlcNAcb1-3Galb1-4(Fuca1-3)GlcNAcb1-3Galb1-4(Fuca1-3)GlcNAcb-Sp0	Tri-Lewis^y^	50
**692/29**	381	Fuca1-2Galb1-3(Fuca1-4)GlcNAcb1-3(Galb1-4GlcNAcb1-6)Galb1-4Glc-Sp21	Extended Lewis^b^	45
**2-25 LE**	57	Fuca1-2Galb1-3(Fuca1-4)GlcNAcb-Sp8	Lewis^b^	**100**
**2-25 LE**	117	Galb1-3(Fuca1-4)GlcNAc-Sp8	Lewis^a^	92
**2-25 LE**	118	Galb1-3(Fuca1-4)GlcNAcb-Sp8	Lewis^a^	90
**BR96**	71	Fucα1-2Galβ1-4(Fucα1-3)GlcNAcβ-Sp0	Lewis^y^	**100**
**BR96**	69	Fucα1-2Galβ1-4(Fucα1-3)GlcNAcβ1-3Galβ1-4(Fucα1-3)GlcNAcβ-Sp0	Lewis^yx^	94
**BR96**	72	Fucα1-2Galβ1-4(Fucα1-3)GlcNAcβ-Sp8	Lewis^y^	87

### Binding Characteristics of Anti-Lewis^y^ and Lewis^b^ mAbs on a Range of Cell Lines

**Figure 2:**
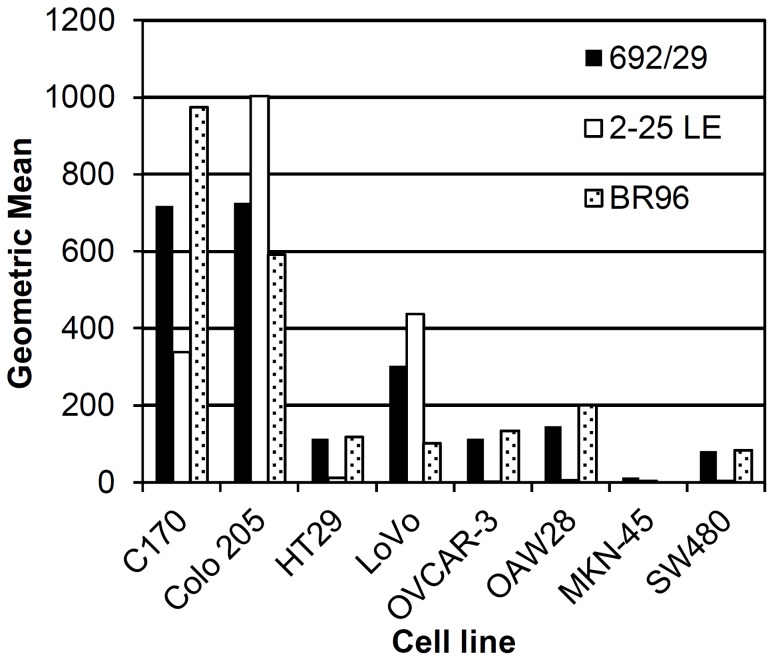
Binding of anti-Lewis^y^ and Lewis^b^ mAbs to a panel of tumour cell lines. A range of cancer cell lines were stained by indirect immunofluorescence with mAbs 692/29, BR96 and 2-25 LE and analysed by flow cytometry. An IgG isotype control was used as a negative control. This assay was repeated 6 times and one representative result is shown.

In order to establish whether the difference in the fine specificity on the glycan array between the three mAbs, related to a difference in binding characteristics, 692/29 (Lewis^y/b^), 2-25 LE (Lewis^b^) and BR96 (Lewis^y^) were screened against a range of cell lines (Fig. 2). Titrations with each mAb were carried out against each cell line with no prozone effect observed at higher concentrations (data not shown). Therefore a therapeutically relevant concentration of 10 µg/ml was chosen for binding assays. 692/29 and BR96 bound all of the cell lines, with the exception of MKN-45 cells, but at varying levels. BR96 stained C170 more strongly whereas 692/29 stained Colo 205 more strongly. 2-25 LE stained C170, Colo 205 and LoVo but failed to stain HT29, OVCAR-3, OAW28, MKN-45 and SW480.

### Induction of ADCC and CDC in Cancer Cells by 692/29, BR96 and 2-25 LE

**Figure 3:**
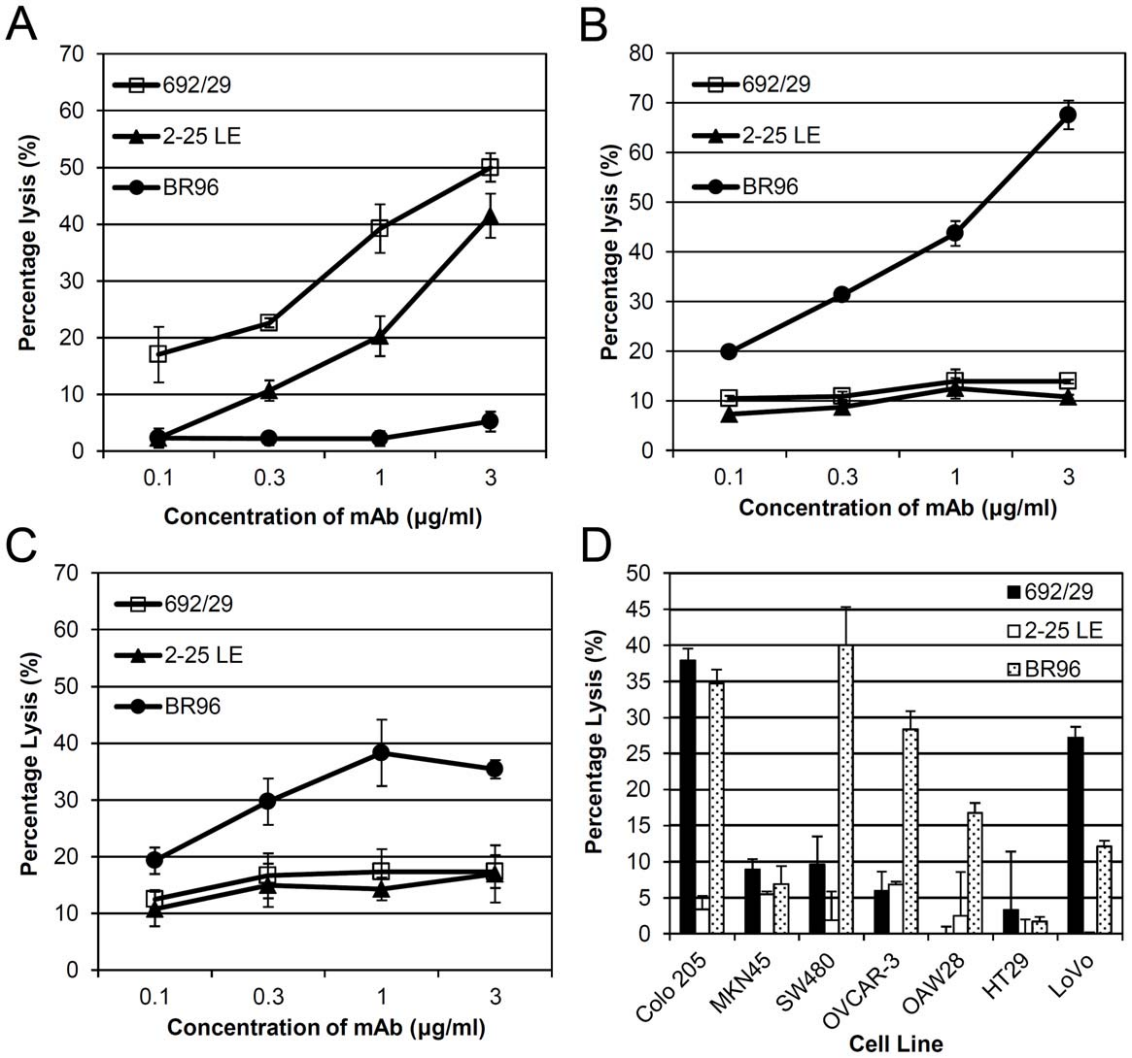
ADCC and CDC assays with anti-Lewis^y^ and Lewis^b^ mAbs. Anti-Lewis^y^ and Lewis^b^ mAbs, 692/29 2-25 LE and BR96 were screened by chromium release assay for ADCC with human PBMCs on Colo 205 (A), OVCAR-3 (B), OAW28 (C) and for CDC lysis with human serum (D). All data points have error bars, but may be obscured by marker. The assay was repeated 3 times and one representative result is shown.

Figure 3 shows that 692/29 was able to induce ADCC in a cell line that displayed a high level of binding (Colo 205; Fig. 3A), but was unable to induce ADCC in cells with lower levels of binding (OVCAR-3, OAW28; Fig. 3B and 3C respectively). 2-25LE is an IgG_1_, which we would not expect to see any significant ADCC or CDC. Despite this, 2-25 LE was able to induce ADCC in Colo 205 cells. BR96 was able to induce ADCC in OAW28, and OVCAR-3 cells despite the fact that it bound quite weakly to these cell lines. Interestingly, despite binding strongly to Colo 205, BR96 failed to induce ADCC in this cell line suggesting that ADCC mediated by the other 2 mAbs was mediated through Lewis^b^.

Figure 3D shows that 692/29 was able to induce CDC in Colo 205 and LoVo cells but not in the weak binding cell lines; SW480, OVCAR3, OAW28, MKN-45 or HT29. In contrast, BR96 was able to induce CDC in Colo 205, LoVo, OAW28, SW480, and OVCAR-3, despite the fact that it bound quite weakly to the latter two lines. It did, however, fail to induce CDC in the very low and negative cell lines HT29 and MKN-45. As expected, the IgG_1_ 2-25 LE was unable to induce CDC in any of the cell lines tested.

### Induction of Cell Death in Colorectal Cancer Cells by 692/29, BR96 and not 2-25 LE

**Figure 4:**
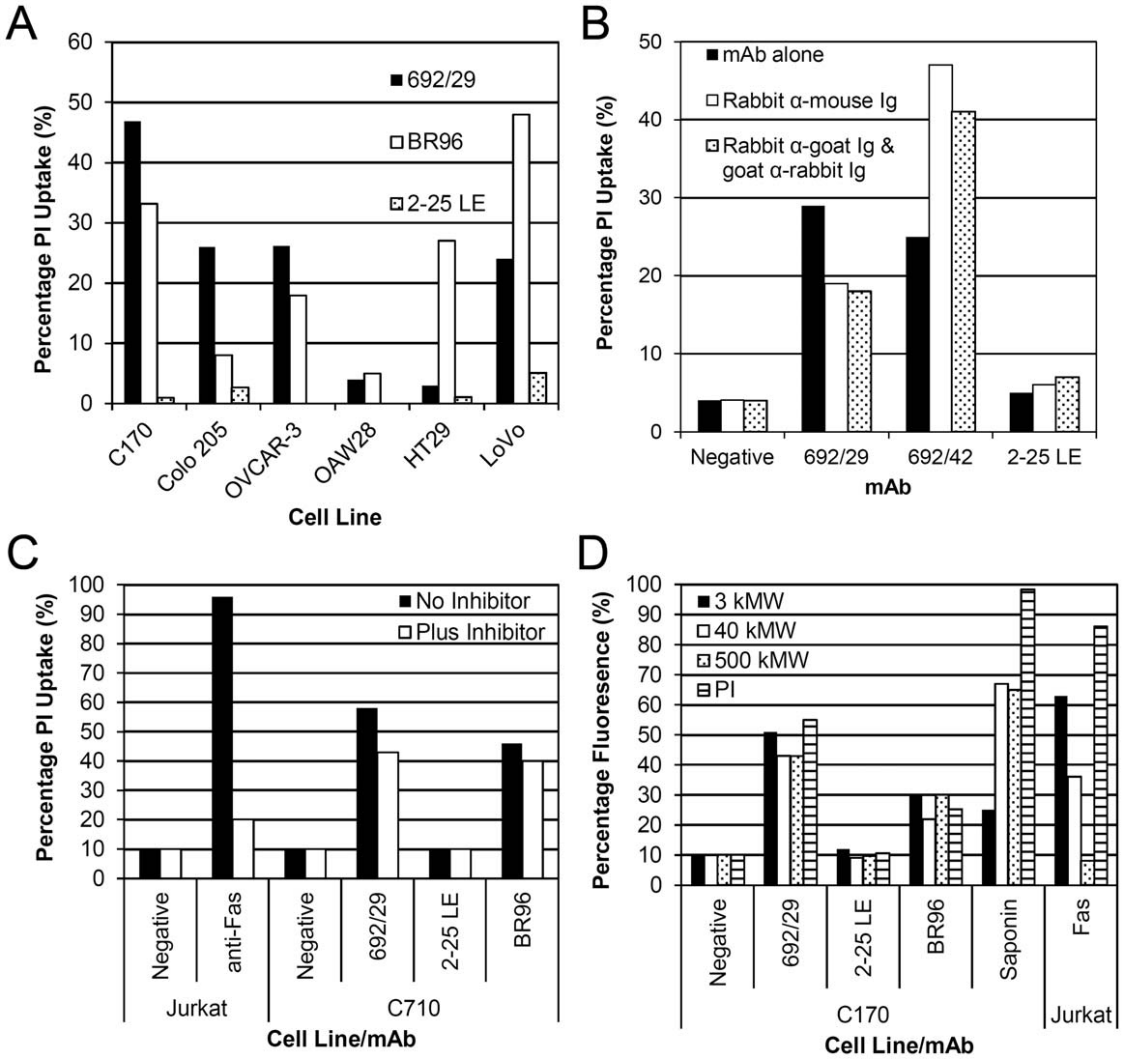
Direct killing assay with anti-Lewis^y^ and Lewis^b^ mAbs. Anti-Lewis^y^ and Lewis^b^ mAbs, 692/29 2-25 LE and BR96 were screened for direct killing by uptake of PI and flow cytometric analysis. (A) A range of cell lines were incubated with 692/29, BR96, 2-25 LE or an isotype control overnight at 37°C. (B) C170 cells were incubated with 30 µg/ml 692/29, 692/42 and 2-25 LE either alone, or with rabbit anti-mouse or rabbit anti-mouse and goat anti-rabbit mAbs overnight at 37°C. (C) C170 cells were incubated with 30 µg/ml 692/29, 2-25 LE and BR96 with and without a pan caspase inhibitor overnight at 37°C. Jurkat cells were incubated with 500 µg/ml anti-Fas as a positive control. (D) C170 cells were incubated with 30 µg/ml mAb and 3, 400 and 500 K MW dextran-FITC beads or PI overnight at 37°C before measuring uptake using flow cytometry. Treatment with 0.4% saponin was used as a positive control for bead uptake. Anti-Fas treatment of Jurkat cells was used as a control for apoptosis (negative control for 500 kMW dextran uptake). This assay was repeated 3 times and one representative result is shown.

To determine if the anti-Lewis mAbs could induce direct killing they were incubated on a panel of cells lines which show differential binding. Membrane perturbation was assessed using propidium iodide (PI) uptake (Fig. 4A). 692/29 induced PI uptake in C170, Colo 205 and LoVo. Little PI uptake was observed in cells with low levels of Lewis^y/b^ expression; HT29, and OAW28 cells (Fig. 4A). However, good PI uptake was seen in OVCAR3 despite low levels of antigen expression, suggesting that this cell line was very susceptible to killing. BR96 induced PI uptake in C170, Colo 205, OVCAR-3, HT29 and LoVo cells, despite binding weakly to HT29 (Fig. 4A). 2-25 LE failed to induce PI uptake in any of the cell lines, despite showing strong binding to C170 and Colo 205 (Fig. 4A). Histograms of PI uptake are shown for C170 and summarised as percentage uptake (Fig. S1). In order to confirm PI uptake corresponded to cell death C170 cells were incubated with 692/29 for 5 days. Initially the number of cells decreased overnight before continuing to grow at the same rate as untreated cells. After 5 days, the surviving cells expressed similar levels of antigen to untreated cells (data not shown).

2-25 LE demonstrated highest binding to Colo 205, and LoVo cell lines, but failed to induce PI uptake and cell death. Both 692/29 and BR96 are IgG_3_ mAbs, which have been reported to be able to dimerise and cross-link on the cell surface, whereas 2-25 LE is an IgG_1_. In order to determine whether antibody dimerisation was important for the killing effect, 2-25 LE was cross-linked with isotype specific secondary antibodies A cell line bound by all three mAbs (C170) was incubated with 2-25 LE in the presence and absence of rabbit anti-mouse and goat anti-rabbit mAbs, providing a cross-linked lattice on the cells. As a positive control an IgG_1_ class switch variant of 692/29 (692/42) was used. This anti-Lewis^y/b^ has been previously shown to cause PI uptake in Lewis^y/b^ positive cell lines that is increased with the addition of cross-linking antibodies (unpublished data). Figure 4B shows PI uptake induced by 692/42 was increased with the addition of cross-linking antibodies. However, despite cross-linking 2-25 LE, no PI uptake was observed.

To further investigate the mechanism of direct cell killing mediated by 692/29 and BR96, they were incubated with cells in the presence or absence of a pan caspase inhibitor, Z-FMK-VAD. Figure 4C shows that despite the addition of 20 mM Z-FMK-VAD, no inhibition of 692/29 and BR96-mediated cell death was observed. To investigate if the cell death was mediated on metabolically active cells, mAb induced death was measured after incubation of C170 cells at 22°C and 4°C. Figure S2 shows that 692/29 and BR96 could induce uptake of PI at both 22 and 4°C, whereas incubation of C170 cells with 2-25 LE at 22 and 4°C did not. Apoptosis in Jurkat cells induced by anti-Fas was inhibited partially at 22°C and completely at 4°C (Fig. S2).

The induction of PI uptake in the presence of a pan caspase inhibitor and at both 22 and 4°C, suggested that the mechanism of direct cell death was not mediated in a caspase-dependent manner, but may be related to oncosis or other forms of caspase-independent cell death. Oncosis is associated with plasma membrane damage, caused by the formation of large pores in the membrane. This is similar to detergent lysis, so saponin is used as a positive control. Oncosis is in contrast to apoptosis, which only allows uptake of small molecular weight drugs such as PI, which is why anti-Fas is used as a negative control. Uptake of FITC-conjugated dextran beads ranging from 3 kMW to 500 kMW in size were used to study the size of pore formation induced by 692/29 and BR96. Figure 4D shows that the 3, 40 and 500 kMW dextran beads were able to diffuse into the 692/29 and BR96 treated cells, leading to an increased level of fluorescence when compared to the untreated and 2-25 LE treated controls. This suggests that the size of pores formed on the cell surface by 692/29 and BR96 are large enough to permit uptake of beads of 500 kMW. In contrast, the anti-Fas mAb on Jurkat cells, which induced apoptosis only allowed PI uptake.

### 692/29, BR96 and 2-25 LE Binding to Normal and Tumour TMAs

**Figure 5:**
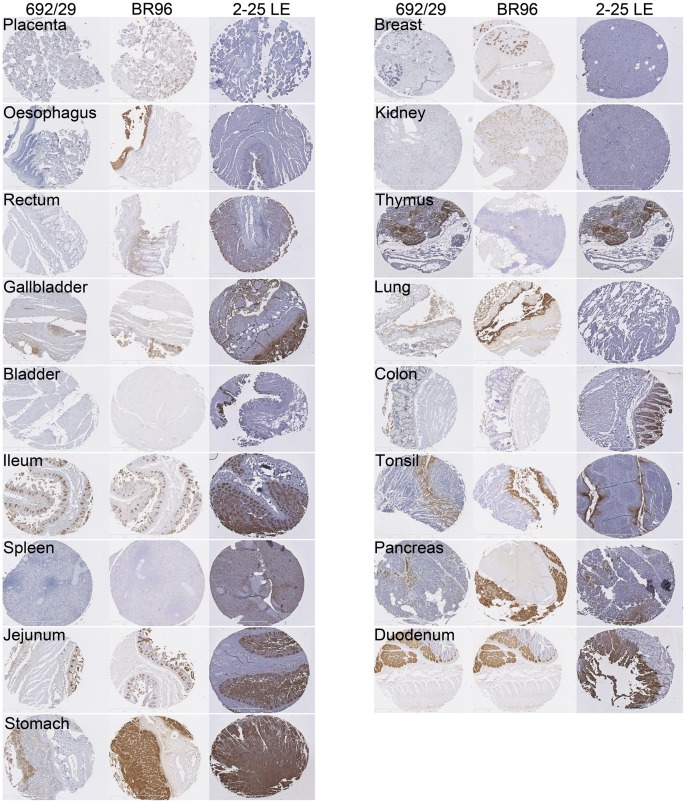
Binding of 692/29, BR96 and 2-25 LE to normal tissues. 692/29, BR96 and 2-25 LE were assessed for normal tissue binding by IHC. Normal staining to placenta, oesophagus, rectum, gallbladder, bladder, ileum, spleen, jejunum, stomach, breast, kidney, thymus, lung, colon, tonsil, pancreas and duodenum are represented for 692/29 (A), BR96 (B) and 2-25 LE (C).

As the 692/29 mAb has been shown to bind to both Lewis^y^ and Lewis^b^ haptens, it was important to determine the specificity and normal tissue reactivity of the mAb in comparison with the more specific Lewis^y^ and Lewis^b^ mAbs BR96 and 2-25 LE (Table 2 and Fig. 5). 692/29 stained one tissue; jejunum strongly, four tissues; stomach, ileum, pancreas and tonsil moderately and five tissues weakly; gallbladder, thymus, lung, duodenum and colon. Twenty one tissues failed to stain. In contrast, BR96 stained nine tissues strongly; oesophagus, ileum, jejunum, stomach, breast, lung, tonsil, pancreas and duodenum; two tissues moderately: gallbladder and placenta and three tissues weakly; rectum, kidney and colon. Seventeen tissues failed to stain. 2-25 LE stained four tissues strongly; bladder, ileum, jejunum and stomach, seven tissues moderately; rectum, gallbladder, thymus, colon, tonsil, duodenum and pancreas and two tissues weakly; spleen and breast. Eighteen tissues failed to stain.

**Table 2:** Binding of 692/29 to normal tissues compared to BR96 and 2-25 LE. No binding, weak, moderate or strong binding represented by 0, 1, 2, 3 respectively.

Tissue type	Antibody		
	692/29	BR96	2-25LE
Placenta	0	2	0
Oesophagus	0	3	0
Rectum	0	1	2
Gallbladder	1	2	2
Skin	0	0	0
Adipose	0	0	0
Heart	0	0	0
Skeletal	0	0	0
Bladder	0	0	3
Ileum	2	3	3
Spleen	0	0	1
Brain	0	0	0
Jejunum	3	3	3
Stomach	2	3	3
Breast	0	3	1
Kidney	0	1	0
Testis	0	0	0
Cerebellum	0	0	0
Liver	0	0	0
Thymus	1	0	2
Cervix	0	0	0
Lung	1	3	0
Smooth Muscle	0	0	0
Colon	1	1	2
Ovary	0	0	0
Tonsil	2	3	2
Diaphragm	0	0	0
Pancreas	2	3	2
Uterus	0	0	0
Duodenum	1	3	2
Thyroid	0	0	0

Both 692/29 and BR96 displayed a good distribution on colorectal tumours, binding by 692/29 and BR96 was seen on 82% and 90% of tumours respectively, with 39% and 49% of these tumours staining moderately or strongly. 2-25 LE showed a poorer distribution, binding only 52% of colorectal tumours with 19% staining moderately and none staining strongly (Table 3, Fig. S3).

**Table 3:** Binding of mAbs to a colorectal tumour microarray. Binding of mAbs to a colorectal tumour array was assessed. Levels of expression are shown as percentage of tumours bound.

Antibody	Intensity of staining			
	Negative	Weak	Moderate	Strong
692/29	17.4	43.5	23.9	15.2
BR96	9.3	41.9	27.9	20.9
2-25 LE	47.6	33.3	19.1	0.0

### *In vivo* Studies

**Figure 6:**
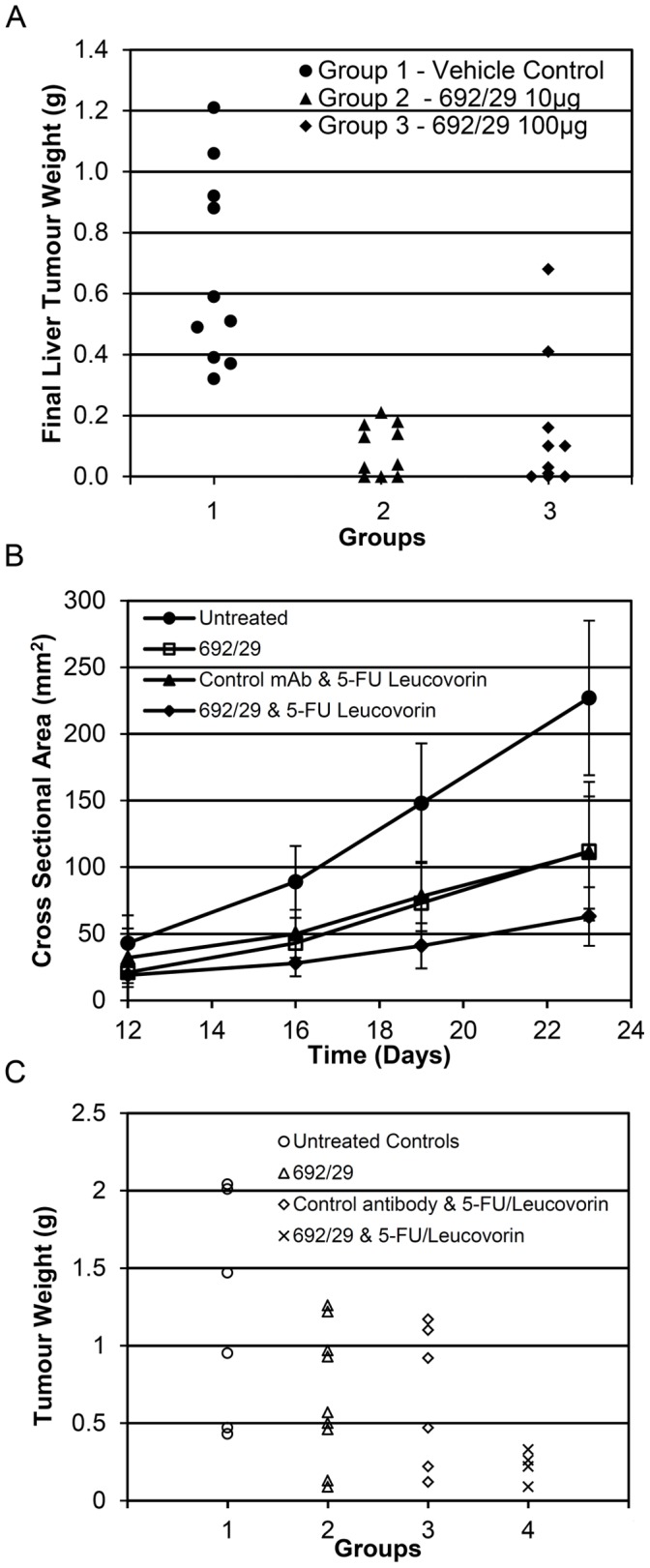
692/29 *in vivo* killing. (A) The effect of 10 µg or 100 µg 692/29 mAb, or the vehicle control, on the final liver tumour weights (g) in the C170HM2 liver metastases nude mouse model. The median values for 10 µg and 100 µg of 692/29 mAb (Groups 2 and 3) were found to be significant (p<0.001) from vehicle control (Group 1). (B) The effect of 692/29 mAb, 5-FU/leucovorin or a combination of 692/29 mAb and 5-FU/leucovorin on the growth of C170 xenografts growing in nude mice. Growth of C170 xenografts was measured at days 12, 16, 19 and 23 by measurement of cross-sectional area (mm^2^) when animals were treated with either 692/29 mAb ip (0.2 mg), control antibody ip (0.2 mg) and 5-FU/leucovorin (12.5 mg/kg; iv). Analysis of variance of the results from day 23 showed the significant values of p<0.004 when comparing 692/29 mAb to the untreated control group and p<0.020 when comparing 692/29 mAb plus 5-FU/leucovorin to 5-FU/leucovorin with control antibody but due to the variability in tumour growth this did not reach significance when compared to 692/29 mAb alone. (C) Termination tumour weights of mice not treated (group 1), immunised with 692/29 mAb (group 2) alone, 5-FU/leucovorin alone (group 3) or the combination of 692/29 mAb and 5-FU/leucovorin (group 4). The combination of 692/29 mAb and 5-FU/leucovorin showed significantly lower tumour weights when compared to control (P>0.01) or to either 692/29 mAb or 5-FU/leucovorin on their own (p<0.05).

692/29 mAb was initially tested *in vivo* in a therapeutic liver metastases model (C170HM2). Mice were treated with the two doses of 100 µg and 10 µg of 692/29 mAb three times weekly for 6 weeks following injection of tumour cells. At the termination of the study the weights of the excised C170HM2 liver tumour were measured. The median for each group was calculated and the statistical significance of these assessed using the Mann-Whitney Test (Fig. 6A). The values for 10 µg and 100 µg 692/29 mAb (Groups 2 and 3) were found to be significantly less (p<0.001) than the vehicle control (Group 1).

A second therapeutic model was assessed where antibody was administered 3 times weekly to mice 5 days after being inoculated with C170 tumour cells or after receiving 3 mm^2^ explants of C170 tumours. In this series 692/29 mAb treatment was compared to standard chemotherapy (5-FU/leucovorin) or a combination of both for 3 weeks. Figure 6B shows that both 692/29 mAb and 5-FU/leucovorin alone resulted in a similar inhibition of tumour growth (p<0.004 Anova). In addition, the combination of both showed additive inhibition of growth with only 2/10 mice showing any growth above the 0.3 g weight of the implanted tumours (Fig. 6C, p<0.02 Anova). At this dose 692/29 mAb was well tolerated, with all mice showing no loss of weight or any other gross pathology (data not shown). The doses and schedule were designed to be therapeutically relevant, with the schedule based on the clearance kinetics of IgG in mice and doubling time of tumours *in vivo* and dosing based on levels achievable in a clinical setting.

## Discussion

Lewis antigens have been proven to be over-expressed on a range of tumours making them good tumour markers [1], [2], [3], [5], [6], [17], [18]. MAbs recognising these haptens were, therefore, assessed for their therapeutic potential. In this study we describe the characterisation of a mAb, 692/29, that binds to both Lewis^y^ and Lewis^b^ haptens, shows good tumour specificity and potent anti-tumour activity *in vitro* and *in vivo*. Additionally, we demonstrate that differences in antigen specificity impacts on *in vitro* cancer cell distribution and function.

High-throughput carbohydrate microarray profiling of 27 anti-glycan mAbs demonstrated widespread specificity problems with several antibodies cross reacting with a range of other blood group antigens that are expressed on a range of normal tissues [19]. This is also true for mAbs targeting Lewis^y^ as they consistently cross-react with Lewis^x^ and H-type 2 structures. This can lead to undesirable cross-reactivity with normal tissues leading to subsequent toxicities in clinical trials. For example, in a phase I study of murine mAb BR55-2, which cross-reacts with H antigen, there was haematuria in 6/12 patients and diarrhoea in 2/9 patients with only transient reductions in skin lesions seen in 3 patients [20]. MAbs BR96 and hu3S193 are more Lewis^y^ specific but in clinical studies gastrointestinal binding was dose limiting and shown to be related to antibody binding [11], [13]. In contrast, Lewis^b^ is expressed at low levels by the surface epithelium and is also upregulated in tumours [1], [21], but no mAbs have entered clinical trials.

The specificity of binding of 692/29 and BR96 was confirmed on the Consortium for Functional Glycomics glycan array. These results were compared to array binding data for 2-25 LE obtained from the Consortium database. The array confirmed that BR96 bound to its described antigen, Lewis^y^, but also reacted with a range of Lewis^y^ variants (Lewis^yx^, Lewis^yxx^) and more weakly to Lewis^x^ and that 2-25 LE bound to Lewis^b^, with cross-reactivity with Lewis^a^ and Lewis^ax^. 629/29 bound most strongly to Lewis^b^ and glycans containing Lewis^b^ and also to tri-Lewis^y^ and its variants. This is an interesting feature as Lewis^y^ or Lewis^b^ mAbs have previously been described, including BR96 and 2-25 LE, but only one mAb has been reported to bind both antigens [22]. However, this mAb has haemagglutinating properties suggesting it cross reacts with H antigen and so would not be useful therapeutically [22]. Structural analysis of the anti-Lewis^y^ mAbs BR96 and hu3S193 has shown that the mAbs have an extremely similar mechanism of binding to Lewis^y^, with no cross-reactivity with Lewis^b^, suggesting structural convergence of immune recognition of Lewis^y^ [22]. 692/29 has previously been shown to bind to both Lewis^y^ and Lewis^b^ on a high percentage of ovarian, colorectal, and gastric cancers [9]. In order to observe whether the differences in fine specificity of the mAbs influenced binding to cancer cells, the mAbs were screened against a range of colorectal and ovarian cancer cell lines. This revealed differing binding between the mAbs, suggesting that difference in specificity of the mAbs may affect their *in vivo* distribution.

BR96 has previously been shown to induce cell death, both directly and by immune-mediated mechanisms. To establish whether the difference in specificity of mAbs has an effect on their ability to induce cell death, their *in vitro* killing mechanisms were compared. It would have been preferable to compare these antibodies with the same isotypes i.e. all IgG3’s as this isotype promotes both ADCC and CDC. This was not possible and is one of the limitations of the immune effector results. 692/29 (IgG3) was a potent inducer of complement lysis, ADCC and direct tumour killing. Of great interest was that the killing was restricted to those cells that displayed higher levels of the Lewis^y/b^ antigen. In contrast, BR96 (IgG3) induced killing of cells expressing high and lower levels of antigen with the exception of Colo 205. The lack of killing of Colo205 by BR96 may be due to its binding to Lewis^x^ and not Lewis^y^ on these cells, as studies have shown that the cell line Colo 205 does not express Lewis^y^ [23]. 2-25 LE did not display any ADCC or CDC as expected of mouse IgG1 mabs with human effector cells and complement. It did kill Colo 205 cells by ADCC, presumably due to the high antigen density giving high avidity and overcoming the low affinity of IgG_1_ for human FcRγIII. A mouse IgG3 2-25LE may have given greater ADCC or CDC, however, this would not have altered the direct killing ability of 2-25LE as it failed to kill even when cross linked. As the direct killing, synergies with ADCC and CDC, even if 2-25LE had been isotype switched it would still be inferior to 692/29 and BR96. None of the antibodies induced antigen clustering during CDC.

As both Lewis^y^ and Lewis^b^ are expressed at low levels on normal cells and the haptens on these tissues are less well recognised by 692/29 this would predict a better therapeutic index for 692/29 than BR96. The mechanism of direct cytotoxicity induced by the mAbs was investigated by incubation with a range of cell lines and observing the level of PI uptake. Both BR96 and 692/29 had the ability to induce PI uptake in cells whereas 2-25 LE did not. BR96 was able to induce PI uptake in cells where it showed weak binding, whereas, 692/29 was only able to induce PI uptake in cells with a high expression of Lewis^y/b^. Previous studies had shown that BR96 was able to induce rapid cell death due to mAb recycling [24]. A proportion of 692/29 also internalises, but mAb is still available on the cell surface for immune mediated cytotoxic effects. It was suggested that BR96 mediated direct cell death this was not caused by apoptosis. This is consistent with our observation that the killing was not inhibited by a pan caspase inhibitor or by incubation of cells with mAbs at 4°C. Studies have shown that at temperatures lower than 15°C Bax is unable to insert into the mitochondrial membrane, inhibiting its ability to cause cytochrome C release and therefore the activation of caspases in neutrophils [25]. MAbs have been shown to induce the caspase-independent cell death pathway, oncosis. For example, RAV12, which binds the carbohydrate antigen RAAG12, binds 90% of intra-abdominal tumours and has been shown to induce oncosis and inhibit growth of tumour xenografts [26]. A phase I study in 33 recurrent adenocarcinoma patients showed some anti-tumour activity of RAV12, although toxicity of the mAb precluded the delivery of maximal doses [27]. The anti-porimin mAb, has been shown to cause oncosis in Jurkat cells [28], while MAb 84, that binds to a glycoprotein, has been shown to induce pore formation and oncosis in human embryonic stem cells using a range of different sized FITC-conjugated dextran beads [29]. More recently, sera from patients treated with an anti-idiotype mAb mimicking the GSL NeuGc-GM3, has the ability to induce the production of human anti-Neu5Gc-GM3 mAbs that induce oncosis in a leukemic cell line [30]. One of the hallmarks of oncosis is the formation of large pores that are unique to oncosis and not seen in apoptosis. Both 692/29 and BR96 mAbs induced pores large enough to allow 500 kMW dextran beads into the treated cells, suggesting that both BR96 and 692/29 have the ability to mediate oncosis in Lewis^y^ positive cells. As the mAbs tested are murine, it will be important to ensure that chimeric or humanised versions of 692/29 maintain their cytotoxic effects. A good indication is that the chimeric version of BR96 displayed cytotoxic effect by inducing direct cytotoxicity, ADCC and CDC [31]. Furthermore, the chimeric BR96 gave the strongest anti-tumour effect in xenograft models when compared to the mouse BR96 [31].

To compare the therapeutic value of 692/29, BR96 and 2-25 LE, the binding of the mAbs to normal and colorectal cancer tissue was assessed. Because 692/29 recognises both Lewis^y^ and Lewis^b^ it may have been predicted that 692/29 would show strong cross-reactivity with a range of normal tissues expressing either Lewis^y^ or Lewis^b^. However, 692/29 showed a reduced cross-reactivity with normal tissues when compared to BR96 and 2-25 LE. MAbs BR96 and hu3S193 are more Lewis^y^ specific but in clinical studies upper gastrointestinal binding was dose limiting and shown to be related to antibody binding [11], [13]. As 692/29 bound more weakly to stomach, pancreas, duodenum, ileum and gallbladder than Br96 and failed to kill cells with low antigen binding, it may be less toxic. It did, however, show similar strong binding to the jejunum.The more restricted normal binding may be that the unique facet of both Lewis^y^ and Lewis^b^ which is recognised by 692/29 is not exposed/accessible on all normal tissues. One possible explanation is that BR96 has a higher affinity than 692/29. This could also explain the greater level of cytotoxicity induced by BR96 in cell lines with low levels of antigen. Thus, the possible lower affinity seems to provide 692/29 with a better therapeutic window. As both antibodies show non saturation binding kinetics, it is difficult to measure affinity. Both 692/29 and BR96 bound to a high proportion of colorectal tumours, with 2-25 LE binding to a limited proportion of tumours. Due to the good *in vitro* distribution and functionality observed with 692/29 against colorectal tumours, its *in vivo* ability was assessed. 692/29 inhibited the growth of C170 colorectal tumour xenografts and prevented liver metastases. In combination with 5-FU/leucovorin it was even able to control the growth of established tumours. This is comparable to *in vivo* data for the murine BR96, which displayed an anti-tumour effect against a lung carcinoma model [31].

In conclusion, our study suggests that mAbs directed at the same antigen, display subtle differences in binding *in vitro*, which may affect tissue distribution *in vivo* and are dependent upon their fine specificity. This can lead to the improved therapeutic benefit of mAbs recognising more than one glycan. 692/29 is a novel mAb that recognises Lewis^y/b^ and shows minimal cross-reactivity with normal tissues whilst binding to tumours. 692/29 induced inhibition of cell growth which was further enhanced in combination with chemotherapy. Therefore, 692/29 could be used in combination with these drugs to reduce toxicity and increase efficacy of treatment for cancer.

## Materials and Methods

### Ethics Statement

Ethical approval was given to use archived human tumour samples and to produce a database by the Nottinghamshire Local Research Ethics Committee. Patient consent was not taken as the materials were all annoymised prior to use in the study. All steps have been taken to ameliorate animal suffering during this study. All work was carried out under a Home Office License and in accordance with Home Office Animal Welfare Guidelines. The study was approved by Nottingham University institutional review board.

### Cell Lines

C170 is a colorectal cell line derived from primary tumours [2]. Colo 205, HT29, SW480, and LoVo are colorectal adenocarcinoma cell lines, OVCAR-3 and OAW28 are both ovarian cancer cell lines, MKN-45 is a gastric adenocarcinoma cell line and Jurkat is a cell line derived from a T cell leukaemia, which were all obtained from ATCC (Virginia, USA). C170HM2 is a colorectal cell line that metastasises to the liver, originally derived from a primary tumour [32]. All cells were cultured in RPMI 1640 medium (BE12-702F; Cambrex Bio Science, Berkshire, UK) supplemented with 10% foetal calf serum (FCS F6178; Sigma, Poole, UK).

### Monoclonal Antibodies

The 692/29 mAb and BR96 (HB 10036 hybridoma; ATCC) were purified as described previously [7], [9]. The anti-Lewis^b^ mAb, 2-25 LE was purchased from Abcam (Cambridge, UK) and the IgG isotype control from Sigma (Sigma, Dorset, UK). All hybridomas were cultured in RPMI, supplemented with 10% foetal calf serum (Sigma) and maintained at 37°C and 5% CO_2_. Human activating anti-Fas receptor mAb (clone CH11) was purchased from Millipore (MA, USA).

### Glycan Screening

To determine the fine specificity of 692/29 and BR96, the mAbs were FITC labelled and sent to the Consortium for Functional Glycomics Core H, where they were screened against versions 3.2 (406 glycans) and 4.1 (465 glycans) respectively, of the printed array with glycans in replicates of 6. 2-25 LE glycan array binding data to version 3.0 of the array was obtained from the Consortium for Functional Glycomics Core H database.

### Flow Cytometry Analysis

C170, Colo 205, HT29, LoVo, OVCAR-3, OAW28, MKN-45 and SW480 cells were plated at 1×10^5^ per well and incubated for 1 hr at 4°C with 10 µg/ml 692/29, BR96 or 2-25 LE (diluted in 10% FCS/RPMI). The cells were then washed with phosphate buffered saline (PBS; Oxoid) and incubated at 4°C for 30 min with rabbit anti-mouse IgG FITC (Dako, Glostrup, Denmark; diluted 1∶100 in 10% FCS/RPMI). Excess FITC was washed off and the cells were subsequently analysed by flow cytometry on a Beckman Coulter FC500 (FL1; Beckman Coulter, CA, USA).

### ADCC

Colo 205, OVCAR-3 and OAW28 cells (2×10^6^) were labelled with (0.925 MBq^51^[Cr] radionuclide) using 200 µCi sodium chromate (PerkinElmer Inc, MA) for 1 hr at 37°C. Peripheral blood mononuclear cells (PBMCs) were isolated from a fresh human blood sample on the same day as the experiment. PBMCs were serially plated into a polystyrene round-bottomed microtiter plate (Nunc) in 10% FCS/RPMI. Assays were started by adding target cells (50 µl), resulting in a final volume of 200 µl and a starting effector-to-target (E/T) cell ratio of 100∶1 unless otherwise indicated. After 20 hrs at 37°C, 50 µl from the top of each well was transferred into a 96-well LumaPlate (Perkin Elmer LAS Ltd) and left to dry overnight. ^51^Cr release from triplicates was measured in counts per minute (cpm). The ^51^Cr released into the medium was counted in a gamma scintillation counter. Percentage of cellular cytotoxicity was calculated with the following formula:
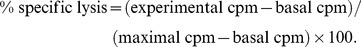



Maximal ^51^Cr release determined by adding Triton X100 (Sigma; 10% final concentration) to target cells and basal release measured in the absence of mAbs and effector cells.

### CDC

5×10^3^ ^51^Cr-labelled target cells per well were coated with the Lewis mAbs and incubated on a 96-well plate with human serum (at variable concentrations) in a total volume of 200 µl. Serum was diluted in RPMI (v/v). In the controls, either mAbs or serum were omitted (spontaneous ^51^Cr release), or a human IgG control was used. The cells were incubated for 16hrs at 37°C. 50 µl of supernatant was taken from the top of each well, transferred into a 96-well LumaPlate (Perkin Elmer LAS Ltd) and left to dry overnight. ^51^Cr release was then calculated as for the ADCC assays.

### Propidium Iodide Uptake Assays

5×10^4^ C170, Colo 205, OVCAR-3, OAW28, HT29 and LoVo cells were incubated at room temperature overnight with 30 µg/ml 692/29, BR96 or 2-25 LE in 2.5% newborn calf serum (NBCS)/RPMI. 0.1 µg PI was added and incubated for a further 30 min. Cells were then harvested and analysed by flow cytometry.

### Cross-linking 2-25 LE mAb and Analysis of PI Uptake

5×10^4^ C170 cells were incubated at room temperature with 30 µg/ml 692/29, 692/42 or 2-25 LE alone, with rabbit anti-mouse Ig or rabbit anti-goat then goat anti-rabbit Igs, overnight in 2.5% NBCS/RPMI. 0.1 µg PI was then added for a further 30 min prior to harvesting and analysed by flow cytometry.

### Uptake of PI Upon mAb Treatment at 4 and 22°C

5×10^4^ C170 cells were incubated with 30 µg/ml 692/29, 2-25 LE or BR96 at room temperature or 4°C for 2 hrs in 2.5% NBCS/RPMI. As a measure of apoptosis, 5×10^4^ Jurkat cells were incubated with anti-Fas (human activating) mAb (Clone CH11, Millipore, Billerica, MA, USA) at 500 ng/ml overnight at 37°C (diluted in 2.5% NBCS/RPMI). Cells were then incubated for a further 30 min with 0.1 µg PI before being harvested and analysed by flow cytometry.

### Estimation of Pore Size with Dextran Bead Uptake

FITC-labelled dextran beads of 3 k, 40 k and 500 kMW were used to estimate the size of pore formation on mAb treated cells. C170 or Jurkat cells were incubated with 30 µg/ml 692/29, 2-25 LE and BR96, 500 ng/ml anti-Fas or 0.5% saponin (Sigma). 0.1 mg FITC-labelled dextran beads or 0.1 µg PI were added to the cells and incubated overnight at 37°C (diluted in 2.5% NBCS/RPMI). Cells were washed twice with PBS to remove excess dextran-FITC before harvesting and analysing cell fluorescence by flow cytometry.

### Analysis of mAb Killing with Pan Caspase Inhibitor

5×10^4^ C170 cells were incubated with 30 µg/ml with or without the pan caspase inhibitor Z-FMK-VAD (20 mM; BD Biosciences, NJ, USA) overnight at 37°C in 2.5% NBCS/RPMI. As a positive control, Jurkat cells were incubated with 500 ng/ml anti-Fas with or without the pan caspase-inhibitor. 30 min prior to the end of the incubation 0.1 µg PI was added to the cells before analysing by flow cytometry.

### Antibody Binding to Tumour and Normal TMAs

A paraffin-embedded array of 31 normal tissues in duplicate was purchased from AMS Biotechnology (Abingdon, UK). The colorectal tumour array, produced in house and previously described, was used to analyse tumour binding [33], [34], [35], [36]. Immunohistochemical analysis of 692/29, BR96 and 2-25 LE binding on both arrays was performed using a routine streptavidin-biotin peroxidase method. Normal array sections were first deparaffinised with xylene, rehydrated through graded alcohol and immersed in methanol containing 0.3% hydrogen peroxide for 20 min to block endogenous peroxidase activity. In order to block non-specific binding of the primary antibody, sections were then treated with 100 µl of 1/50 normal swine serum (NSS; Vector Labs, UK) in PBS for 20 min. Also colorectal and gastric sections were treated with avidin biotin block (Vector Labs) for 15 min. Sections were subsequently incubated with 100 µl of 1 µg/ml 692/29, 5 µg/ml BR96 or 10 µg/ml *2*-25 LE NSS/PBS for 1 hr at room temperature. Following incubation of the sections with 100 µl of biotinylated goat anti-mouse/rabbit immunoglobulin (Vector Labs, UK) diluted 1∶50 in NSS, for 30 min, they were washed in PBS. They were then incubated with 100 µl of pre-formed streptavidin-biotin/horseradish peroxidase (HRP) complex (Vector Labs) for 30 min at room temperature. Subsequently, visualisation of 692/29 and BR96 expression was achieved using 3, 3′-Diaminobenzidine tetra hydrochloride (DAB, Dako Ltd). Finally, sections were lightly counterstained with haematoxylin (Vector Labs), dehydrated in alcohol, cleared in xylene (GentaMedica, York, UK) and mounted with distyrene, plasticizer and xylene (DPX; BDH, Poole, UK).

### *In vivo* Studies

#### 1. Metastases model

The C170HM2 cells were maintained *in vitro* in 10% heat inactivated FCS-supplemented RPMI 1640 culture medium at 37°C in 5% CO_2_, humidified conditions. Cells from sub-confluent monolayers were harvested with 0.025% EDTA, washed twice in the culture medium outlined above and re-suspended for *in vivo* administration, in sterile PBS, pH 7.4. 1.5×10^6^ cells, in a volume of 1 ml/mouse, were injected into the peritoneal cavity of 30 male nude mice. Animals were allocated to their treatment groups and treatment began on day 1 and continued throughout the study. The groups of mice were treated with either 10 µg or 100 µg of 692/29 mAb or the vehicle control by intravenous (iv) injection on day 1 and then 3 times weekly. Mice were terminated on day 40 and body and tumour weight evaluated.

#### 2. Prevention model

The colorectal tumour cell line, C170 was maintained in serial passage in nude mice. For therapy the mice were sacrificed and the tumours excised. The tumour was finely minced and 3 mm^2^ pieces were implanted, under anaesthetic (Hypnorm, Roche/Hypnovel, Jannsen) subcutaneously, into 40 male mice which had been randomly allocated to 4 experimental groups. A group of mice were treated with 5-FU/leucovorin (12.5 mg/kg) by iv injection on days 1, 3, 5, 7, 21 and 22. Three times weekly mice were also injected intraperitoneally (ip) with 0.2 mg of 692/29 mAb. Control mice received 692/29 mAb, 5-FU/leucovorin or vehicle alone. Tumour size was measured by callipers and tumour cross-sectional area calculated on days 12, 16, 19 and 23. At the termination of the experiment tumours were weighed to assess anti-tumour efficacy. Animals were weighed to assess the toxicity of treatment.

#### 3. Therapeutic model

The colorectal tumour cell line, C170 was maintained in serial passage in nude mice. For therapy the mice were sacrificed and the tumours excised. The tumour was finely minced and 3 mm^3^ pieces were implanted, under anaesthetic, subcutaneously into 40 male mice which had been randomly allocated to 4 experimental groups. Groups of C170 xenografted mice were treated with 5-FU/leucovorin (25 mg/kg) by iv injection on days 1, 3, 5, 7 and cycled, where applicable, from day 28. Three times weekly mice were also injected iv with 692/29 mAb (0.2 mg), control mice receiving either 692/29 mAb alone or control mouse IgG antibody with 5-FU/leucovorin.

### Statistics

Statistical analysis was performed using Analysis of Variance and Log Rank on the Minitab programme for the PC.

## Supporting Information

Figure S1 Histograms depicting uptake of PI after 692/29, BR96 and 2-25 LE treatment of C170 cells. 5×10^4^ C170 cells were incubated with an isotype control (A), BR96 (B), 2-25 LE (C) or 30 µg/ml 692/29 (D) overnight at 37°C. PI was added and uptake was measured by flow cytometry.
(TIF)

Figure S2 Uptake of PI after 692/29, BR96 and 2-25 LE treatment at 4 and 22°C. C170 cells were incubated with 30 µg/ml 692/29, BR96, 2-25 LE or an isotype control and Jurkat cells were incubated with 500 ng/ml anti-Fas mAb overnight at 4°C and 22°C. PI was added and uptake measured by flow cytometry.
(TIF)

Figure S3 Colorectal tumour cores stained with 692/29, BR96 and 2-25 LE. A colorectal TMA was stained with 692/29 (A), BR96 (B) and 2-25 LE (C) and binding was assessed. Examples of weak, moderate and strong binding are shown for each mAb (left to right), with no strong example for 2-25 LE as it did not stain any core strongly. All are at X20 original magnification and the inset ruler measures 300 µm.
(TIF)

Table S1 Details of glycan binding by 692/29 (a), 2-25 LE (b) and BR96 (c) to the glycan array. A blue squares represents glucosylamine, yellow circles represents galactose, red triangles represent fucose, green circles represent mannose and purple diamond represents sialic acid. Sp denotes the length of spacer between glycan and slide. Percentage of best binder refers to the level of binding in relation to the glycan bound best by each mAb.
(DOCX)
