# Supplementary figures and images for: Therapeutic Targeting of Lewisy and Lewisb with a Novel Monoclonal Antibody 692/29
Source: PLoS One. 2013 Feb 8;8(2):e54892. doi: 10.1371/journal.pone.0054892 (PMC3568143; doi:10.1371/journal.pone.0054892)

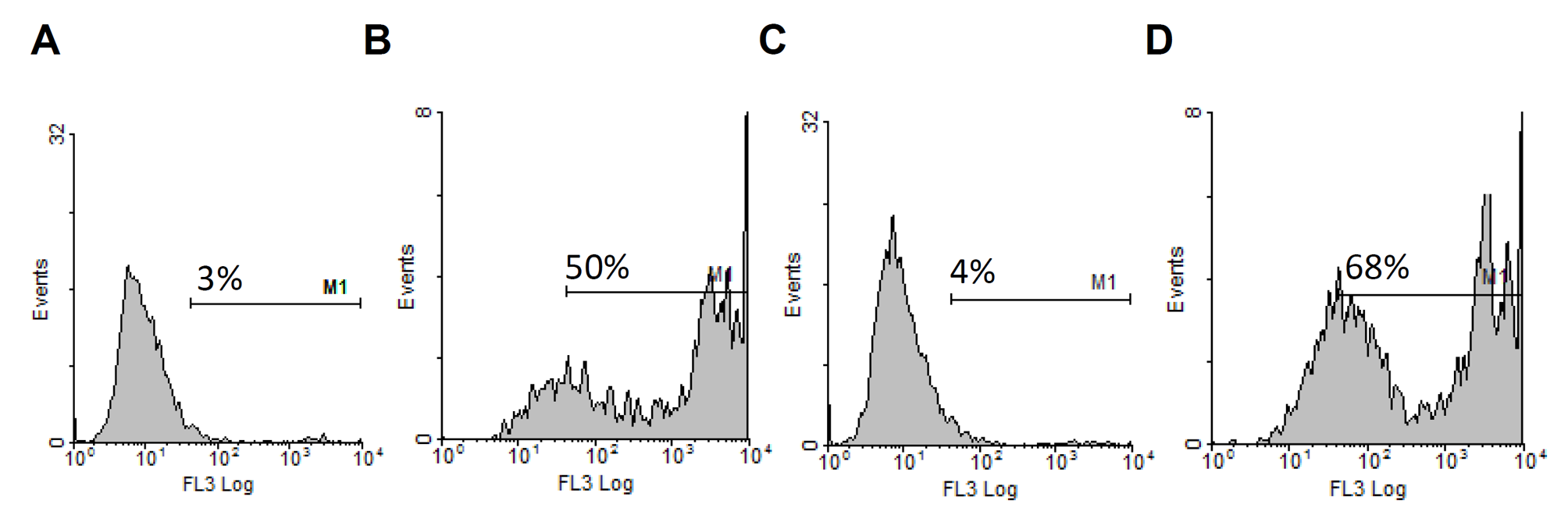

Supplement: Figure S1 — Histograms depicting uptake of PI after 692/29, BR96 and 2-25 LE treatment of C170 cells. 5×10^4^ C170 cells were incubated with an isotype control (A), BR96 (B), 2-25 LE (C) or 30 µg/ml 692/29 (D) overnight at 37°C. PI was added and uptake was measured by flow cytometry. (TIF) [file pone.0054892.s001.tif]

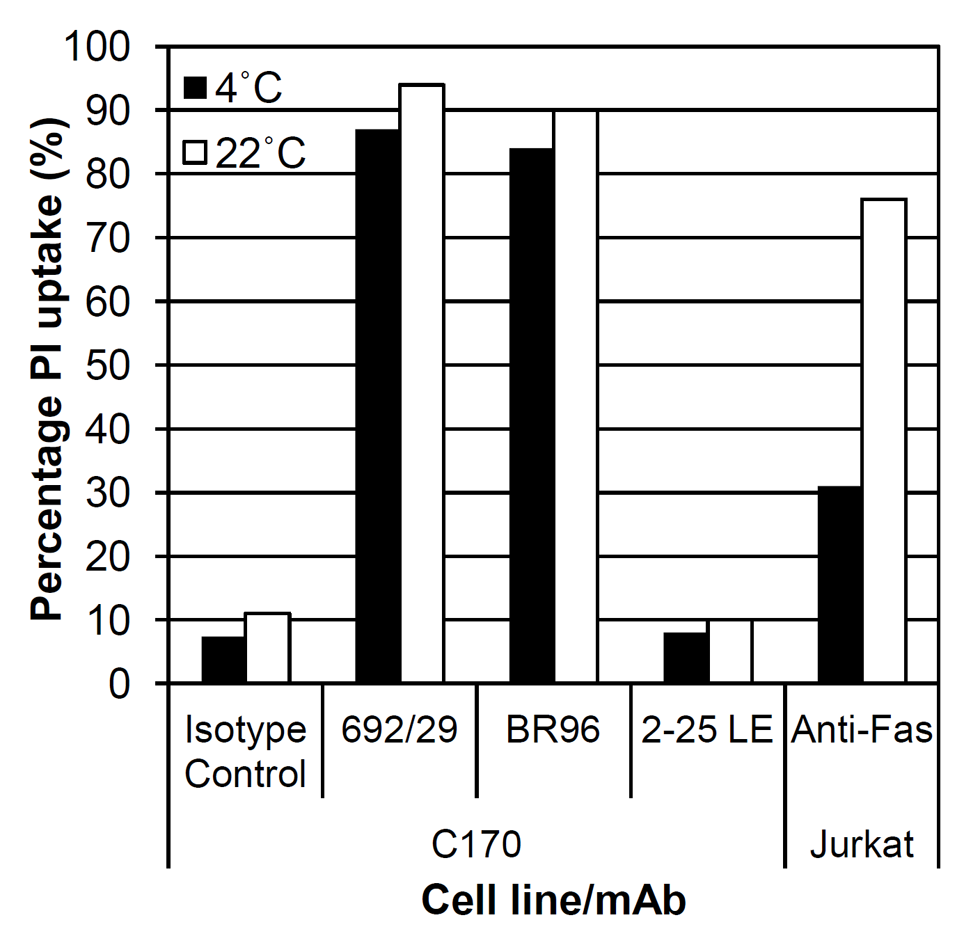

Supplement: Figure S2 — Uptake of PI after 692/29, BR96 and 2-25 LE treatment at 4 and 22°C. C170 cells were incubated with 30 µg/ml 692/29, BR96, 2-25 LE or an isotype control and Jurkat cells were incubated with 500 ng/ml anti-Fas mAb overnight at 4°C and 22°C. PI was added and uptake measured by flow cytometry. (TIF) [file pone.0054892.s002.tif]

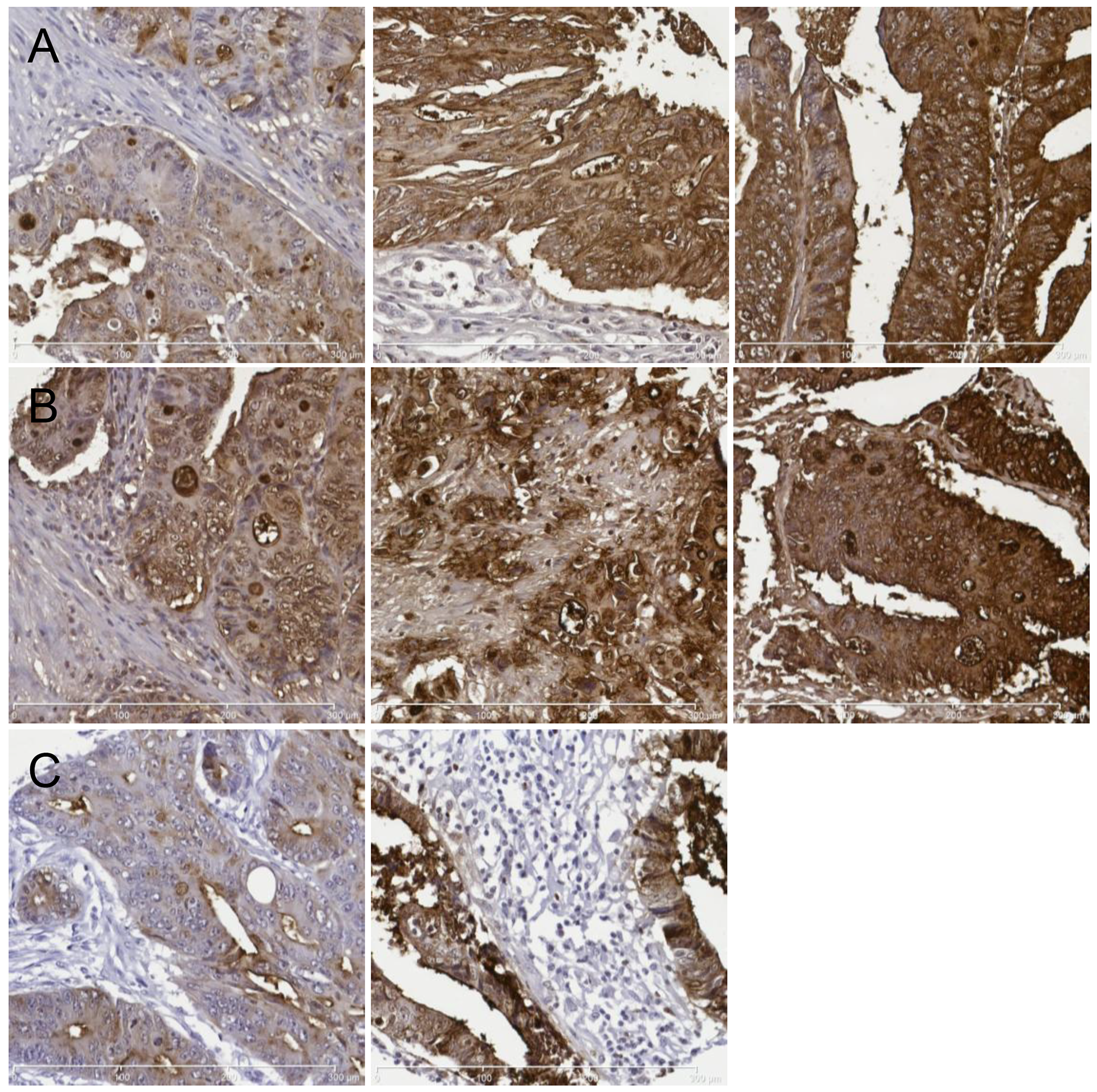

Supplement: Figure S3 — Colorectal tumour cores stained with 692/29, BR96 and 2-25 LE. A colorectal TMA was stained with 692/29 (A), BR96 (B) and 2-25 LE (C) and binding was assessed. Examples of weak, moderate and strong binding are shown for each mAb (left to right), with no strong example for 2-25 LE as it did not stain any core strongly. All are at X20 original magnification and the inset ruler measures 300 µm. (TIF) [file pone.0054892.s003.tif]
