# Supplementary material for: Therapeutic Targeting of Lewisy and Lewisb with a Novel Monoclonal Antibody 692/29
Source: PLoS One. 2013 Feb 8;8(2):e54892. doi: 10.1371/journal.pone.0054892 (PMC3568143; doi:10.1371/journal.pone.0054892)
Supplement: Table S1 — Details of glycan binding by 692/29 (a), 2-25 LE (b) and BR96 (c) to the glycan array. A blue squares represents glucosylamine, yellow circles represents galactose, red triangles represent fucose, green circles represent mannose and purple diamond represents sialic acid. Sp denotes the length of spacer between glycan and slide. Percentage of best binder refers to the level of binding in relation to the glycan bound best by each mAb. (DOCX) [file pone.0054892.s004.docx]

**Table S1 Details of glycan binding by 692/29 (a), 2-25 LE (b) and BR96 (c) to the glycan array.** A blue squares represents glucosylamine, yellow circles represents galactose, red triangles represent fucose, green circles represent mannose and purple diamond represents sialic acid. Sp denotes the length of spacer between glycan and slide. Percentage of best binder refers to the level of binding in relation to the glycan bound best by each mAb.

| **Glycan No.**  **A** | **Glycan** | **Name** | **Percentage of best binder (%)** | **Structure** |
| --- | --- | --- | --- | --- |
| 57 | Fuca1-2Galb1-3(Fuca1-4)GlcNAcb-Sp8 | Lewis b | **100** | 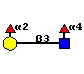 |
| 66 | Fuca1-2Galb1-4(Fuca1-3)GlcNAcb1-3Galb1-4(Fuca1-3)GlcNAcb1-3Galb1-4(Fuca1-3)GlcNAcb-Sp0 | Lewis y-x-x | 50 | 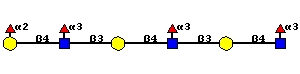 |
| 381 | Fuca1-2Galb1-3(Fuca1-4)GlcNAcb1-3(Galb1-4GlcNAcb1-6)Galb1-4Glc-Sp21 | Lewis x- containing glycan | 45 | 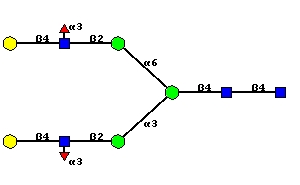 |
| 382 | Fuca1-2Galb1-3(Fuca1-4)GlcNAcb1-3(Galb1-4(Fuca1-3)GlcNAcb1-6)Galb1-4Glc-Sp21 | Lewis b-x containing glycan | 37 | 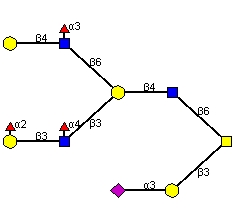 |
| 114 | Galb1-3(Fuca1-4)GlcNAcb1-3Galb1-4(Fuca1-3)GlcNAcb-Sp0 | Lewis a-x | 27 | 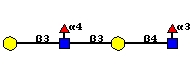 |
| 68 | Fuca1-2Galb1-4(Fuca1-3)GlcNAcb-Sp8 | Lewis y | 26 | 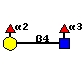 |
| 118 | Galb1-3(Fuca1-4)GlcNAcb-Sp8 | Lewis a | 23 | 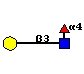 |
| 116 | Galb1-3(Fuca1-4)GlcNAcb-Sp0 | Lewis a | 19 | 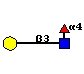 |
| 200 | Fuca1-3(Galb1-4)GlcNAcb1-2Mana1-3(Fuca1-3(Galb1-4)GlcNAcb1-2Mana1-6)Manb1-4GlcNAcb1-4GlcNAcb-Sp20 | Lewis a-containing glycan | 18 | 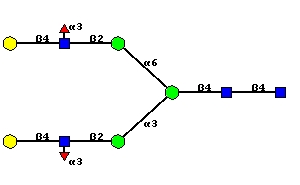 |
| 65 | Fuca1-2Galb1-4(Fuca1-3)GlcNAcb1-3Galb1-4(Fuca1-3)GlcNAcb-Sp0 | Lewis y-x | 14 | 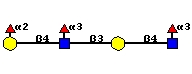 |

| **Glycan No.** | **Glycan** | **Name** | **Percentage of best binder (%)** | **Structure** |
| --- | --- | --- | --- | --- |
| 57 | Fuca1-2Galb1-3(Fuca1-4)GlcNAcb-Sp8 | Lewis b | **100** | 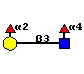 |
| 117 | Galb1-3(Fuca1-4)GlcNAc-Sp8 | Lewis a | 92 | 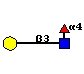 |
| 118 | Galb1-3(Fuca1-4)GlcNAcb-Sp8 | Lewis a | 90 | 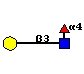 |
| 119 | Galb1-3(Galb1-4GlcNAcb1-6)GalNAca | Type-2 containing glycan | 84 | 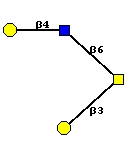 |
| 114 | Galb1-3(Fuca1-4)GlcNAcb1-3Galb1-4(Fuca1-3)GlcNAcb-Sp0 | Lewis a-x | 79 | 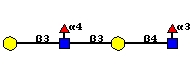 |
| 274 | Galb1-3(Neu5Aca2-3Galb1-4GlcNacb1-6)GalNAca-Sp14 | Sialylated Type-2 containing glycan | 48 | 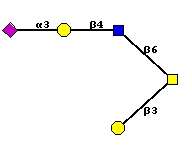 |
| 217 | Neu5Aca2-3Galb1-3(Fuca1-4)GlcNAcb1-3Galb1-4(Fuca1-3)GlcNAcb-Sp0 | Sialyl Lewis a-x | 40 | 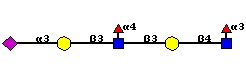 |
| 218 | Galb1-3(Fuca1-4)GlcNAcb-Sp0 | Lewis a | 33 | 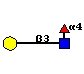 |

**B**

| **Glycan No.**  **C** | **Glycan** | **Name** | **Percentage of best binder (%)** | **Structure** |
| --- | --- | --- | --- | --- |
| 71 | Fucα1-2Galβ1-4(Fucα1-3)GlcNAcβ-Sp0 | Lewis y | **100** | 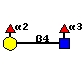 |
| 69 | Fucα1-2Galβ1-4(Fucα1-3)GlcNAcβ1-3Galβ1-4(Fucα1-3)GlcNAcβ-Sp0 | Lewis y-x | 94 | 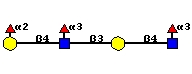 |
| 72 | Fucα1-2Galβ1-4(Fucα1-3)GlcNAcβ-Sp8 | Lewis y | 87 | 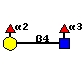 |
| 70 | Fucα1-2Galβ1-4(Fucα1-3)GlcNAcβ1-3Galβ1-4(Fucα1-3)GlcNAcβ1-3Galβ1-4(Fucα1-3)GlcNAcβ-Sp0 | Lewis y | 84 | 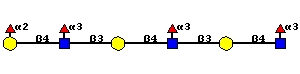 |
| 103 | Galα1-3(Fucα1-2)Galβ1-4(Fucα1-3)GlcNAcβ-Sp0 | Lewis y-x-x | 74 | 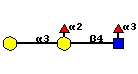 |
| 104 | Galα1-3(Fucα1-2)Galβ1-4(Fucα1-3)GlcNAcβ-Sp8 | Lewis y-x-x | 70 | 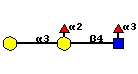 |
| 151 | Galβ1-4(Fucα1-3)GlcNAcβ1-4Galβ1-4(Fucα1-3)GlcNAcβ-Sp0 | Di-Lewis X | 51 | 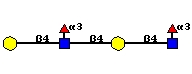 |
| 152 | Galβ1-4(Fucα1-3)GlcNAcβ1-4Galβ1-4(Fucα1-3)GlcNAcβ1-4Galβ1-4(Fucα1-3)GlcNAcβ-Sp0 | Tri-Lewis X | 42 | 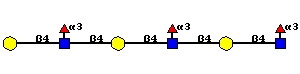 |
| 361 | Fucα1-2Galβ1-4(Fucα1-3)GlcNAcβ1-2Manα1-3(Fucα1-2Galβ1-4(Fucα1-3)GlcNAcβ1-2Manα1-6)Manβ1-4GlcNAcβ1-4GlcNAβ-Sp20 | Extended Lewis y | 16 | 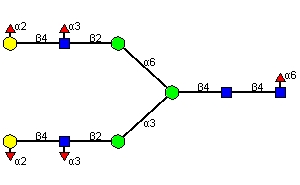 |
| 149 | Galβ1-4(Fucα1-3)GlcNAcβ-Sp0 | Lewis X | 7 | 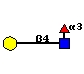 |
| 446 | Fucα1-2Galβ1-4(Fucα1-3)GlcNAcβ1-2(Fucα1-2Galβ1-4(Fucα1-3)GlcNAcβ1-4)Manα1-3(Fucα1-2Galβ1-4(Fucα1-3)GlcNAcβ1-2Manα1-6)Manβ1-4GlcNAcβ1-4GlcNAcβ-Sp12 | Extended Lewis y | 6 | 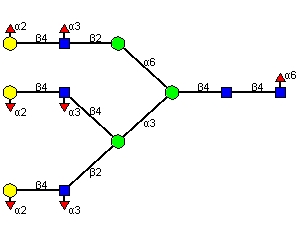 |
